# Bacteriological profile of wound infections and antimicrobial resistance in selected gram-negative bacteria

**DOI:** 10.4314/ahs.v22i4.63

**Published:** 2022-12

**Authors:** Altaf Bandy, Farooq A Wani, Abdul H Mohammed, Umar F Dar, Ayesha Mallick, Mushtaq R Dar, Bilal A Tantry

**Affiliations:** 1 College of medicine, Jouf University, Sakaka, post code 42421, Saudi Arabia; 2 Ministry of Health, AlJouf Region, Sakaka, post code 42421, Saudi Arabia; 3 Ministry of Interiors, Saudi Arabia

**Keywords:** Wound infections, hospital, Gram-negative bacteria, antibiograms, multidrug-resistance, *E. coli*

## Abstract

**Background:**

Managing wound infections is a challenging task. Understanding their resistance pattern is an essential step at reducing its burden in hospital settings.

**Objective:**

To determine the bacteriological diversity of wound infections and the antimicrobial resistance exhibited by a selected Gram-negative bacterium in the Aljouf region of Saudi Arabia.

**Methods:**

The study retrospectively analysed the antibiograms of wound infections from hospitalized patients for the year 2019. The European Centre for Disease Control guidelines were adopted for the classification of resistant bacteria. Multidrug-, extensive drug-, and carbapenem-resistant isolates are presented as frequencies and percentages.

**Results:**

A total of 295 non-duplicate wound swab antibiograms were retrieved, 64.4% (190) and 35.6% (105) isolates were Gram-negative and Gram-positive bacterial infections respectively. Predominant pathogens included Staphylococcus species 21.0% (62), *E. coli* 16.3% (48) and *K. pneumoniae* 13.5% (40). 148 (77.9%), 42 (22.1%) and 43 (22.6%) of the Gram-negative isolates were multidrug-, extensively drug- and carbapenem-resistant. The antibiotic resistance exhibited by gram-negative bacteria was 43.4% (234/539), 59.1% (224/379) and 53.7% (101/188) towards carbapenems, 3^rd^ - and 4^th^ – generation cephalosporins.

**Conclusions:**

The majority of wound infections are caused by multidrug-, extensively drug- and carbapenem-resistant Gram-negative bacteria. Further studies should focus on the molecular basis of this resistance.

## Introduction

Infectious diseases are a common cause of morbidity and mortality 1. Impairment of the first line of defence especially damage to the skin and mucous membranes facilitate the entry of microorganisms into the human body resulting in infections 2.

Wound infections increase the chances of wound dehiscence and delay healing 3. Traumatic injuries are the most common etiological factor in the genesis of wounds in hospitalized patients. Traumatic injuries are categorized according to the mode of occurrence into accidental and intentionally induced wounds. Hospital-acquired wounds such as surgical incisions or intravenous medical devices are categorized as intentionally induced wounds whereas non-intentionally induced wounds include wounds such as decubitus ulcers 4. The major cause of acquired wound infections in the hospital is surgical interventions. Surgical site infections (SSI) can be allocated into three groups namely superficial incisional SSI, deep incisional SSI, and organ-specific SSI 5.

A high rate of postoperative wound infections has been observed in developing countries^6^–^8^. Post-operative wound infection is exerting huge stress on healthcare because of its morbid and financial implications and has become a major concern in the healthcare settings asking for cost management systems to be adopted^9^. There is an urgent need to formulate surveillance programs for detecting and diagnosing surgical site infections along with the antibiotic susceptibility pattern of infecting organisms in order to reduce the associated complications and morbidity 10.

Wound infections in hospitalized patients are frequently caused by *Staphylococcus aureus, Escherichia coli, coagulase-negative Staphylococcus (CoNS), Pseudomonas aeruginosa, Proteus mirabilis, Enterobacter aerogenes and Klebsiella pneumonia* 11. S. aureus has been the most dominant bacterial isolate reported in most of the studies 4, 12–14. With regards to antimicrobial resistance of the bacterial isolates to multiple antibiotics is concerned, the high prevalence was observed among the gram-negative bacteria 15.

Some of the studies from Saudi Arabia have documented the gram-negative bacteria to be the predominant isolates from wound infections. A study conducted in a teaching hospital in Riyadh have found *E. coli* as the predominant bacteria and observed the highest antibiotic resistance in *Pseudomonas* 16.

An extensive review of PubMed and Google scholar did not reveal any study on the bacteriological profile and antimicrobial resistance patterns of gram-negative bacteria causing wound infections from the Aljouf region of Saudi Arabia. The current study will help us in evaluating the gram-negative spectrum and their resistance patterns in wound infections that will guide infection control measures and anti-microbial stewardship programs.

## Methods

### Setting and design

The present study was conducted in a specialist hospital in Sakaka, the capital city of the Aljouf region the Kingdom of Saudi Arabia. There are two specialist hospitals in Sakaka city which serve as referral hospitals for the Aljouf region. Aljouf region comprises of three governorates of Sakaka, Qurayyat, and Dumat Al-Jandal with a total population of five million and twenty thousand.. In this cross-sectional study, all antibiograms from January 1, 2019, to December 31, 2019, of hospitalized patients were included. Culture and sensitivity reports of all non-duplicate wound swabs of *E. coli, K. pneumoniae, P. aeruginosa and A. baumannii*, were specifically analysed for antimicrobial resistance.

### Bacterial identification and antimicrobial resistance classification

For the purpose of bacterial identification and antimicrobial sensitivity testing, an automated BD Phoenix system (BD Biosciences, Franklin Lakes, NJ, USA) was used. Clinical and Laboratory Standard Institute recommendations were employed for the antimicrobial susceptibility testing (AST)^17^. We classified resistant microorganisms based on the European Centre for Disease Control; guidelines into multidrug-resistant (MDR), extensive drug-resistant (XDR), and pan drug-resistant (PDR) 18. Intermediate-resistant strains were merged with the resistant strains for simplification of the results. The phenotypic characterization of carbapenem, potential carbapenem, and ESBL producers as provided by the Phoenix system was also recorded. Carbapenem and potential carbapenem producers were categorized as carbapenem producers. All the details regarding the demographic data and hospitalization data were extracted from the hospital records. STROBE-AMS guidelines were adopted to report antimicrobial resistance.

### Consent and research ethics

The research protocol got approved from the Local Committee of Bio-Ethics at Jouf University (vide no: 03/04/41 dated January 6, 2020). Informed consent was not required for this study; however, it should be noted that before a sample is taken, a verbal consent is ensured by the concerned medical personnel in the presence of the patient's relative as a standing operating procedure. Patient's guardian approval is taken and recorded in the medical files for patients admitted in the intensive care units.

### Statistical analysis

The data were analysed using SPSS version 20.0 for Windows (SPSS, Inc., Chicago, IL, USA). MDR, XDR, PDR, two researchers checked the completeness of data at entry. ESBl- and carbapenem producers' frequencies were calculated. The results are presented as frequencies and percentages.

## Results

**Fig 1:**
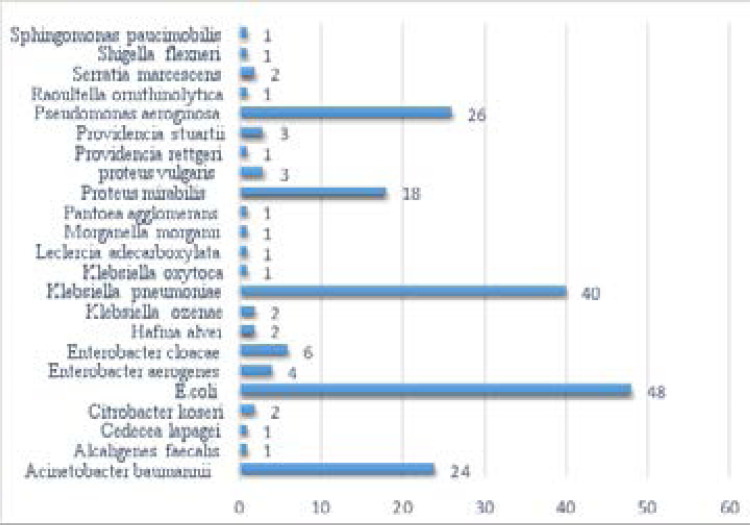
Gram-negative bacterial profile of wound infections

Of the 295 non-duplicate wound swab antibiograms, 190 (64.4%) and 105 (35.6%) were of Gram-negative and Gram-positive bacterial infections. The majority of the samples (57.3%) and (54.6%) were received from male patients and male and female surgical wards. Isolated microorganisms include forty-eight (16.3%) *E. coli*, forty (13.5%) *K. pneumoniae*, twenty-six (8.9%) *P. aeruginosa* and twenty-four (8.1%) as *A. baumannii*. Among the gram-positive bacteria, seventy-six (25.8%) isolates of Staphylococcus species were the most frequent microorganism followed by thirteen (4.4%) isolates of Streptococcus species causing wound infections (Table 1). The Gram-negative profile of wound infections is shown in figure 1.

**Table 1:** Bacterial Profile and sample distribution characteristics of wound Swabs (n=295)

Category	Number (n)	Percentage (%)
**Gram-negative bacteria n= 190 (64.4%)**		
*E. coli*	48	16.3
*K. pneumoniae*	40	13.5
*P. aeruginosa*	26	8.9
*A. baumannii*	24	8.1
*P. mirabilis*	18	6.1
*Enterobacter species*	10	3.4
*P. stuatrii*	3	1.0
Others	21	7.1
**Gram-positive bacteria n= 105 (35.6%)**		
*S. aureus*	62	21.0
*S. epidermedis*	7	2.4
*Other Staphylococcus species*	7	2.4
*Streptococcus species*	13	4.4
*E. faecalis*	8	2.7
Others	8	2.7
* **Quarter**		
Quarter-1	93	31.5
Quarter-2	70	23.7
Quarter-3	53	18.0
Quarter-4	79	26.8
**Referring unit**		
Male & female surgical, Burn and Orthopaedic Wards	161	54.6
Male & female Intensive care units	90	30.5
Referred from other hospitals	23	7.8
Male & female medical wards	21	7.1
**Gender**		
Males	169	57.3
Females	126	42.7
**Age**		
≥60 years	74	25.0
40–59 years	90	30.5
20–39 years	76	25.8
≤19 years	55	18.7

* Year is divided in to quarters, each quarter represents three months e.g., Quarter-1 starts from janurary-1 2019 to March 31^st^ 2019 and so on.

Analysis of the distribution of Gram-negative wound infections revealed that the majority occurred among men (56.3%) and during the first quarter (34.8%) of the year 2019. The male and female surgical, burn and orthopaedic wards contributed to 51.6% of these isolations followed by male and female intensive care units (42.7%). More than 55% of these infections occurred among patients aged 40 years and above. *E. coli* (25.3%), *K. pneumoniae* (21.0%) were the frequent Gram-negative isolates. Resistance pattern showed 148 (77.9%) multi-drug-, 42 (22.1%) extensively drug-, 43 (22.6%) Carbapenem-resistant and 24 (12.6%) isolates were ESBL producers respectively (Table 2).

**Table 2:** Distribution of Gram-negative wound infections and their resistance pattern (n=190)

Category	Number (n)	Percentage (%)
**Gender**		
Males	107	56.3
Females	83	43.7
**Quarter**		
Quarter-1	66	34.8
Quarter-2	47	24.8
Quarter-3	29	15.3
Quarter-4	48	25.3
**Referring unit**		
Male & female surgical, Burn and Orthopaedic Wards	98	51.6
Male & female Intensive care units	81	42.7
Male & female medical wards	11	5.7
**Age**		
≥60 years	49	25.8
40–59 years	57	30.0
20–39 years	49	25.8
≤19 years	35	18.4
**Microorganisms**		
**E. coli**	48	25.3
**K. pneumoniae**	40	21.0
**P. aeruginosa**	26	13.7
**A. baumannii**	24	12.7
**P.mirabilis**	18	9.4
**Enterobacter species**	10	5.3
**P. stuatrii**	3	1.6
Others	21	11.0
**Resistance pattern**		
*MDR	148	77.9
**XDR	42	22.1
***PDR	5	2.6
Carbapenem producer	43	22.6
****ESBL producers	24	12.6

* MDR= Multidrug-resistance

** XDR= Extended drug-resistance

*** PDR= Pan drug-resistance

**** ESBL= Extended spectrum beta lactamases

Overall, the multi-drug (54.7%) and carbapenem-resistant (60.5%) strains were isolated from male patients and the majority of these strains infected patients aged 40years and above. The multidrug- and extensively drug-resistant strains were observed in male and female intensive care unit's unit with a frequency of 45.3% and 66.7% (Table 3).

**Table 3:** Distribution of MDR*, XDR** and Carbapenem resistant strains

Characteristic		MDR (148)	XDR (42)	CP*** (43)
**Gender**	Male	81 (54.7)	22 (52.4)	26 (60.5)
	Female	47 (45.3)	20 (47.6)	17 (39.5)
**Age groups**	≥60 years	38 (25.7)	13 (31.0)	10 (23.3)
	40–59 years	46 (31.0)	10 (23.8)	15 (34.8)
	20–39 years	35 (23.6)	10 (23.8)	11 (25.6)
	≤19 years	29 (19.6)	9 (21.4)	7 (16.3)
**Referring Unit**	Male intensive care unit	37 (25.0)	15 (35.7)	16 (37.2)
	Female intensive care unit	30 (20.3)	13 (31.0)	8 (18.6)
	Male surgical ward	27 (21.6)	5 (11.9)	8 (18.6)
	Female surgical ward	22 (14.9)	4 (9.5)	6 (14.0)
	Burn wards	11 (7.4)	1(2.4)	1 (2.3)
	Male and Female medical wards	10 (6.7)	2 (4.8)	4 (9.3)
	Orthopedics ward	6 (4.0)	2 (4.8)	0 (0)
**Microorganisms**	*E. coli*	30 (20.3)	2 (4.8)	7 (16.3)
	*K. pneumoniae*	27 (18.2)	8 (19.0)	16 (37.2)
	*P. aeruginosa*	26 (17.6)	4 (9.5)	0 (0)
	*A. baumannii*	24 (16.2)	21 (50.0)	0 (0)
	*P. mirabilis*	16 (10.8)	3 (7.1)	8 (18.6)
	*Enterobacter species*	7 (4.7)	2 (4.8)	4 (9.3)
	*P. stuatrii*	2 (2.0)	1 (2.4)	1 (2.3)
	Others	14 (13.2)	0 (0)	7 (16.3)

* MDR= Multidrug- resistance

** XDR= Extended drug-resistance

*** CR= Carbapenem producer

Further analysis of the studied microorganism revealed that 15/48 (31.3%) isolates of *E. coli* and 5/40 (12.5%) of *K. pneumoniae* isolates were ESBL producers. All the recovered isolates (100%) of *P. aeruginosa* and *A. baumannii* were MDR, while as, 67.5% and 62.5% of K. pneumoniae and E. coli were MDR. Furthermore, 87.5% of *A. baumannii* were extensively drug-resistant and 40% of *K. pneumoniae* were carbapenem-resistant (Table 4).

**Table 4:** Proportion of MDR*, XDR** and Carbapenem-resistant strains of the studied Gram-negative organisms (n=138)

Name of the microorganism	MDR [n (%)]	XDR [n (%)]	PDR*** [n (%)]	ESBL producer**** [n (%)]	Carbapenem producer/ Resistant [n (%)]
*E. coli* (48)	30 (62.5)	2 (4.2)	7 (14.6)	15 (31.3)	7 (14.6)
*K.* *pneumoniae* (40)	27 (67.5)	8 (20.0)	3 (7.5)	5 (12.5)	16 (40.0)
*P.* *aeruginosa* (26)	26 (100.0)	4 (15.3)	0 (0)	0 (0)	0 (0)
*A.* *baumannii* (24)	24 (100.0)	21 (87.5)	0 (0)	0 (0)	0 (0)

* MDR= Multidrug-resistance

** XDR= Extended drug-resistance

*** PDR=Pan drug-resistance

**** ESBL= Extended spectrum beta lactamases

The overall antibiotic resistance rate for Gram-negative bacteria was 43.4%, 59.1% and 53.7% towards carbapenems, 3rd - and 4th – generation cephalosporins. Among aminoglycosides, amikacin continues to remain effective against *E. coli, P. aeruginosa* and *K. pneumoniae* with a sensitivity rate of > 98%, > 92% and > 75%. All four organisms under study showed a resistance rate of > 75% for 1st -generation cephalosporins. *E. coli, K. pneumoniae* and *A. baumannii* show a resistance rate of > 45%, >55% and >98% resistance against the 3rd-generation cephalosporins. *A. baumannii* isolates were resistant (>95%) to carbapenems. Colistin was highly effective (>90%) against all the tested isolates of gram-negative bacteria under study. Few isolates of *A. baumannii and P. aeruginosa* that were tested for tigecycline presented 100% resistance (Table 5).

**Table 5:** Antibiotic resistance profiles of *E. coli, K. pneumoniae, P. aeruginosa* and *A. baumannii* (n=138)

Antibiotic	Overall resistance		*E. coli (48)*		*K. pneumoniae (40)*		*P. aeruginosa (26)*		*A. baumannii* *(24)*	
	n/t	%	n/t	%	n/t	%	n/t	%	n/t	%
Amikacin	46/190	24.2	1/48	2.0	9/40	22.5	2/26	7.7	17/24	70.9
Gentamicin	68/190	35.8	7/48	14.6	9/40	22.5	5/26	19.2	22/24	91.6
Ertapenem	93/186	50.0	6/48	12.5	21/40	52.5	26/26	100.0	23/23	100.0
Imipenem	74/167	44.3	9/46	19.6	21/40	52.5	10/26	38.5	23/24	95.8
Meropenem	67/186	36.0	5/45	11.1	20/40	50.0	6/26	23.0	23/24	95.8
Cephalothin	161/180	89.4	40/44	90.9	29/38	76.3	26/26	100.0	23/23	100.0
Cefuroxime	144/186	77.4	26/48	54.2	27/40	67.5	26/26	100.0	23/23	100.0
Cefoxitin	97/187	51.9	7/48	14.6	20/40	50.0	26/26	100.0	23/23	100.0
Ceftazidime	96/190	50.5	22/48	45.8	22/40	55.0	6/26	23.0	23/24	95.8
Ceftriaxone	128/189	67.7	23/48	47.9	27/40	67.5	26/26	100.0	23/24	95.8
Cefepime	101/188	53.7	22/47	46.8	24/39	61.5	6/26	23.0	23/24	95.8
Aztreonam	104/187	55.6	23/48	47.9	24/40	60.0	10/26	38.5	24/24	100.0
Ampicillin	170/188	90.4	36/48	75.0	39/40	97.5	26/26	100.0	23/23	100.0
Amoxicillin and Clavulanate potassium	141/189	74.6	27/48	56.3	28/40	70.0	26/26	100.0	24/24	100.0
Piptazobactam	66/190	34.7	7/48	14.6	22/40	55.0	6/26	23.0	23/24	95.8
Colistin	23/72	32.0	0/2	0.0	1/11	9.0	0/14	0.0	1/23	4.3
Trimethoprim- Sulfamethoxazole	122/189	65.0	29/48	60.4	23/40	57.5	26/26	100.0	17/24	70.9
Nitrofurantoin	121/177	68.4	8/46	17.4	25/37	67.6	23/23	100.0	24/24	100.0
Ciprofloxacin	90/176	51.1	15/41	36.6	21/39	53.8	7/22	31.8	23/24	95.8
Levofloxacin	83/175	47.4	13/41	31.7	17/39	43.6	7/22	31.8	23/24	95.8
Tigecycline	49/88	55.68	3/16	18.8	14/29	48.3	1/1	100.0	3/3	100.0

## Discussion

The information regarding the bacteriological profile of wound infections will be of immense value in the institution of proper antimicrobial therapy and in guiding the infection control measures 19.

The study comprised an analysis of 295 wound swab antibiograms which revealed the predominance of Gram-negative bacterial isolates comprising 190 (64.4%) cases. The majority of the studies throughout the globe have found gram-positive bacterial isolates to be the predominant ones. We came across a few studies as done by Mohammed et al 2017 that reported gram-negative predominance 14. Furthermore, Gamal et al 2011 observed E. coli as the most common isolate 16. The high numbers of gram-negative isolates in our study may be attributed to the inclusion of hospitalized patients only as it is well-known that hospitalization and the procedures undertaken after hospitalization increase the risk of acquiring gram-negative infections. The other causes may include the regional variations in geographic locations and socioeconomic status of the studied population 20.

The predominance of male patients (57.3%) was observed in our study as has been noted in the majority of the other studies 3, 8, 12, 14. The majority of the wound samples (54.6%) were received from male and female surgical, Burn and Orthopaedic wards (Table 1). Nwankwo et al also found an increased incidence of wound infections in the male and female surgical wards 21.

Our results showed that the Staphylococcus species (25.8%) were the most frequent microorganism followed by *E. coli* (16.3%), *K. pneumoniae* (13.5%), *P. aeruginosa* (8.9%) and *A. baumannii* (8.1%) (Table 1). *S. aureus* was the most common organism isolated in our study which is in line with the other studies 4, 8, 14. The predominance of *E. coli* in the gram-negative isolates has been reported in earlier studies as well 8, 11, 13. [Mulu, 2012 #33]

Further analysis revealed that the majority of Gram-negative infections occurred among men (56.3%). The surgical, burn and Orthopaedic wards comprised around 51.6% of these isolates followed by intensive care units (ICU's) at 42.7% (Table 2). This increased frequency of infections may be attributed to the increased turnover of patients in these wards compared to intensive care units. Increased frequency of infections in males admitted to non-medical wards, has been observed by other researchers 21, 22. Males are generally considered more prone to infections than females because of differences in the immune responses as well as disparity of sex-chromosome-linked genes 23. More than 55% of these infections occurred among patients aged 40 years and above. Similar observations were made by Chang et al and Mulu et al 3, 11. Among the gram-negative bacteria, *E. coli* (25.3%) and *K. pneumoniae* (21.0%) were the frequent Gram-negative isolates (Table 2). The predominance of *E. coli* and *K. pneumoniae* has been observed by Muluye et al, Manyahi J and Sisay et al 12, 24, 25. Resistance pattern showed 148 (77.9%) multidrug-, 42 (22.1%) extensively drug-, 43 (22.6%) Carbapenem-resistant and 24 (12.6%) isolates were ESBL producers respectively (Table 2). A study done by Enwuru et al on Gram-negative isolates from wound swabs found 64% of their isolates had multiple drug resistance^26^. A very high degree of multidrug resistance in the range of 88.5 to 97.4% for gram-negative isolates has been observed in some of the studies 6, 11, 25. The reason for the relatively high degree of resistance is the inclusion of two or more antibiotics for calculating the multidrug resistance whereas we used the three or more antibiotics for calculating the multidrug resistance.

In our study, 22.1% of the cases showed extensive drug resistance which seems to be in line with other similar studies as done by Mulu et al and Muluye et al who found XDR in 22.7% and 20.6% of their cases respectively 11,12. Some of the researchers have found a high degree of extensive drug resistance (>70% of cases) 6, 14. This has been ascribed to the rampant use of antibiotics in these areas. The other reasoned fact that they have employed five or more antibiotics only for calculating extensive drug resistance.

Carbapenem resistance was found in 22.6% of the cases in our study whereas it was 12.5% in the study done by Enwuru et al 26 and <8% in a study done by Kader et al.^27^. A high degree of carbapenem resistance in our study may be ascribed to the increased numbers of Hajj and Umrah pilgrims visiting Saudi Arabia, unrestrained use of antibiotics and prevalence of community-acquired infections 28.

Overall, the multidrug- (54.7%) and carbapenem-resistant (60.5%) strains were isolated from male patients especially in the age group of 40–59 years. The multidrug- and extensively drug-resistant strains were mainly observed in intensive care units (Table 3). Ibrahim et al observed an increased prevalence of MDR strains of gram-negative bacteria among male patients admitted in the intensive care units 29. Similar observations were made by Banerjee et al and Agyepong et al. 30, 31. The increased prevalence in ICU has been attributed to the presence of critically ill patients, increased instrumentation, extensive use of antibiotics, cross infections among patients and inadequate hand hygiene practices of healthcare workers 29.

Analysis of the proportion of multidrug-resistance shown by the studied microorganisms revealed that 100%, 67.5% and 62.5% of *P. aeruginosa, A. baumannii*, and *K. pneumoniae* and *E. coli* were multidrug-resistant respectively (Table 4). Mohammad et al observed MDR in, 100%, 94.1% and 100% of *P. aeruginosa, A. baumannii*, and *K. pneumoniae* and *E. coli* 14. Mulaye et al observed MDR in 100%, 75% and 83.4% of *P. aeruginosa, Klebsiella spp*. and *E. coli* 12. Mama et al observed MDR in 90.9%, 92.9% and 93.0% and of *P. aeruginosa, Klebsiella spp*. and *E. coli* 25. Biadglegne et al observed MDR in 100% of all cases of these three bacteria 6. Mulu et al observed MDR in 100% and 88.9%, of *P. aeruginosa, K. pneumoniae* and *E. coli* respectively 11. A relatively lower MDR, especially for K. pneumoniae and E. coli in our study may be because we used the criteria of three or more antibiotics for MDR calculation whereas most of the other studies have used two or more than two antibiotic criteria.

Extensive drug resistance was mainly shown by *A. baumannii* (87.5%) followed by *K. pneumoniae* (20.0%), while 40% of *K. pneumoniae* isolates were carbapenem-resistant. We did not observe the isolation of *A. baumannii* in most of the studies which we used for comparison except Mohammad et al who found 100% XDR for Acinetobacter spp. and 64.7% of *K. pneumoniae* 14. Mama et al observed extensive drug resistance in 21.4% and 17.2% of *K. pneumoniae* and *E. coli* isolates respectively 25. Muluye et al found XDR of 52.6% and 38.4% in *Enterobacter spp*. and *Pseudomonas spp*. respectively. Increased prevalence of *A. baumannii* in Saudi Arabia has been attributed to the extensive usage of wide-spectrum antimicrobial drugs, serious comorbidities in patients and the complexity of the ICU environments 32.

Regarding the studied microorganism, 22.72% of the *E. coli* and *K. pneumoniae* were ESBL producers out of which 31.3% was contributed by *E. coli* and 12.5% by *K. pneumoniae* (Table-4). Kader et al found that 17% of *E. coli* and *K. pneumoniae* were ESBL producers out of which 19.6% was contributed by *E. coli* and 12.6% by *K. pneumoniae* 27. The overall antibiotic resistance rate for Gram-negative bacteria was 43.4%, 59.1% and 53.7% towards carbapenems, 3rd - and 4th – generation cephalosporin's respectively (Table 5). Among aminoglycosides, amikacin continues to remain effective against *E coli, P. aeruginosa* and *K. pneumoniae* with a sensitivity rate of > 98%, > 92% and > 75% respectively. Gamal et al found 4%, 28.8% and 25% resistance against *E. coli, P. aeruginosa* and *K. pneumoniae* 33. The effectiveness of amikacin has been proved in other studies also 34, 35.

All of the four organisms under study showed a resistance rate of > 75% for 1st -generation cephalosporins (Table 5). A high degree of resistance of *E. coli* and *K. pneumoniae* has also been observed by Mama et al. 25. Biadglegne et al observed 50 -70% resistance of *E. coli, P. aeruginosa* and *K. pneumoniae* for cephalothin 6. *E. coli, K. pneumoniae* and *A. baumannii* showed a resistance rate of > 45%, >55% and >98% resistance against the 3^rd^-generation cephalosporins (Table 5). Mohammad et al found the resistance of 12.5%, 52.9% and 100% for *E. coli, K. pneumoniae* and *A. baumannii* 14. Furthermore, *A. baumannii* isolates were resistant (>95%) to carbapenems. Li et al found 100% resistance of *A. baumannii* to carbapenems 36.

Colistin was highly effective (>90%) against all the tested isolates of gram-negative bacteria under study (Table 5). Tan et al in their study on the in vitro activity of colistin in gram-negative bacteria observed >90% effectiveness of colistin for *E. coli* and *K. pneumoniae* as seen in our study but found 33% resistance for *P. aeruginosa* which is in contrast to our study 37. Somily et al observed that 100 % and 93.9% sensitivity of colistin against *A. baumannii* and *P. aeruginosa* respectively 38.

Few isolates of *A. baumannii* and *P. aeruginosa* that were tested for tigecycline presented 100% resistance (Table 5). Gupta et al found 100% resistance of tigecycline to *P. aeruginosa* as in our study but found only 6.3% resistance for *A. baumannii* which is in contrast to our study 39. Tigecycline has been generally found to be effective against *A. baumannii* with >90% susceptibility whereas it has limited efficacy against *P. aeruginosa* 40. Al-Agamy et al in their study at a hospital in Riyadh, Saudi Arabia, observed 56% resistance of *A. baumannii* against tigecycline which signifies the presence of resistant strains of A. baumannii in Saudi Arabia 41.

This is the first study on wound infections from the northern region of Saudi Arabia that will add to the world literature on antimicrobial resistance. Furthermore, the study focused on selected gram-negative microorganisms of global importance. The limitation of this study rests in the lack of molecular characterization of resistance and a single centre study.

## Conclusion

Wound infections are dominated by Gram-negative organisms with a higher frequency of MDR and carbapenem-resistant isolates that will challenge wound management in the light of limited treatment options. Intensive care patients are at a higher risk of acquiring resistant Gram-negative wound infections necessitating strict infection control activities. The frequent empirical antimicrobial therapy for intensive care patients should be based on the local evidence on the bacteriological profile and their resistance pattern. The study recommends strengthening surveillance activities that will guide the control of wound infection in hospitals. Furthermore, effective implementation of antimicrobial prescription guidelines coupled with patient counselling to adherence of antimicrobial consumption in primary health centres is needed.

## Abbreviations

ESBL: extended-spectrum beta-lactamase
ICU: intensive care unit
MDR: multi-drug resistant
PDR: pan-drug resistant
XDR: extensively-drug resistant (XDR)
CR: carbapenem-resistant
